# Spatial and temporal heterogeneity alter the cost of plasticity in *Pristionchus pacificus*

**DOI:** 10.1371/journal.pcbi.1011823

**Published:** 2024-01-30

**Authors:** Ata Kalirad, Ralf J. Sommer

**Affiliations:** Department for Integrative Evolutionary Biology, Max Planck Institute for Biology Tübingen, Tübingen, Germany; Stony Brook University, UNITED STATES

## Abstract

Phenotypic plasticity, the ability of a single genotype to produce distinct phenotypes under different environmental conditions, has become a leading concept in ecology and evolutionary biology, with the most extreme examples being the formation of alternative phenotypes (polyphenisms). However, several aspects associated with phenotypic plasticity remain controversial, such as the existence of associated costs. While already predicted by some of the pioneers of plasticity research, i.e. Schmalhausen and Bradshaw, experimental and theoretical approaches have provided limited support for the costs of plasticity. In experimental studies, one common restriction is the measurement of all relevant parameters over long time periods. Similarly, theoretical studies rarely use modelling approaches that incorporate specific experimentally-derived fitness parameters. Therefore, the existence of the costs of plasticity remains disputed. Here, we provide an integrative approach to understand the cost of adaptive plasticity and its ecological ramifications, by combining laboratory data from the nematode plasticity model system *Pristionchus pacificus* with a stage-structured population model. Taking advantage of measurements of two isogenic strains grown on two distinct diets, we illustrate how spatial and temporal heterogeneity with regard to the distribution of resources on a metapopulation can alter the outcome of the competition and alleviate the realized cost of plasticity.

## Author summary

The ability of living organisms to express different phenotypes without genetic change (phenotypic plasticity) has fascinated biologists, probably since the establishment of biology as a branch of science. Despite its ubiquity in nature, many aspects of phenotypic plasticity remain unresolved. For instance, it has been suggested that a biological system equipped with phenotypic plasticity would necessarily have to pay a cost in fitness compared to a non-plastic counterpart. In this manuscript, we utilize the laboratory data on the nematode plasticity model system *Pristionchus pacificus* to simulate how the cost of plasticity would manifest itself in a competition between a plastic and a non-plastic organism. This nematode exhibits a predatory mouth under certain environmental conditions, including diet. We show how variation in the distribution of resources in space and time can greatly affect the outcome of competition. Our work illustrates the complexity of predicting the ecological consequences of phenotypic plasticity in a changing world.

## Introduction

The expression of alternative phenotypes by a single genotype in different environments, i.e., phenotypic plasticity or polyphenism, remains a topic of great interest and discussion in both ecology and evolution [1–4]. A plastic organism capable of assuming the form and function fitted to multiple environments could have a considerable advantage in competition against genetically hard-wired competitors. However, intuitively, such adaptive plasticity, given the hypothetical machinery behind it, should incur a cost. This possible cost did not escape the pioneers in the study of plasticity; for example, Bradshaw argued that a case of adaptive plasticity could be selected against if the plastic trait were too costly [5].

**Fig 1:**
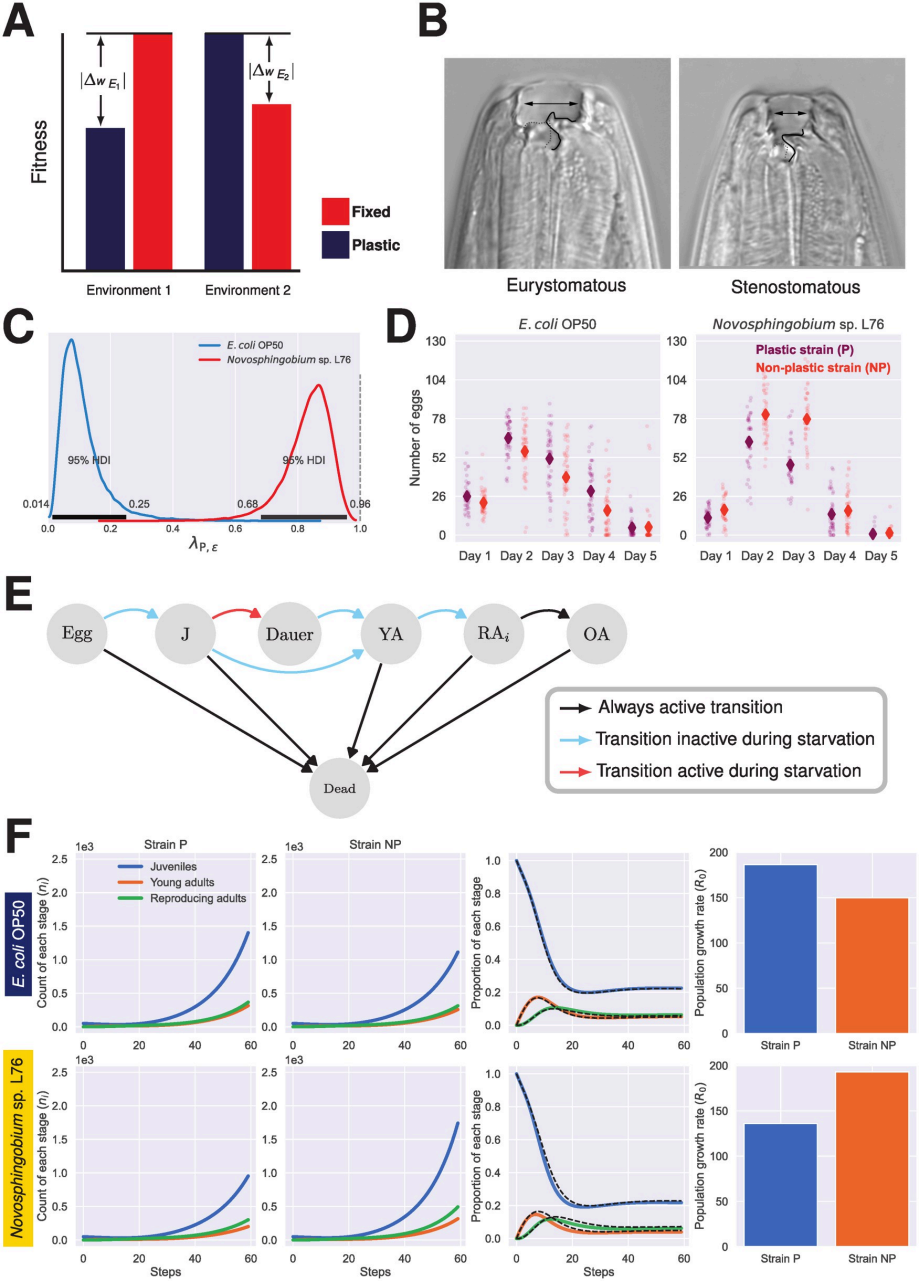
Phenotypic plasticity and associated costs in *P. pacificus*. **(A)** The cost of plasticity in our model can be illustrated in a hypothetical scenario: The plastic strain expresses the defensive phenotype in the presence of predators (environment 1), but this plastic response to environment 1 is accompanied by a reduction in fitness, $\Delta w_{E_{1}}$, which is the cost of plasticity. The fixed strain expresses the defensive phenotype regardless of the presence or the absence of predators. The production of the costly defensive phenotype in the absence of predators (environment 2), resulting in a relative cost of phenotypic mismatch between the fixed genotype and environment 2 ($\Delta w_{E_{2}}$) compared to the fitness of the plastic genotype in this environment. It is evident that costs of phenotype and plasticity are, by definition, exclusively meaningful in comparative studies in environments that can be described as adaptive and non-adaptive with regard to a given trait. The magnitude of the difference between $\Delta w_{E_{1}}$ and $\Delta w_{E_{2}}$ depends on the details of the machinery generating the plastic response, among other factors. Absolute value are used in this schematic figure to avoid any confusion with respect to the sign of these fitness differences. **(B)** The nematode *P. pacificus* expresses two alternative mouth forms, the predatory (eurystomatous) and the non-predatory (stenostomatous) from, in response to a variety of external stimuli. The fate of the mouth form is determined during post-embryonic development. **(C)** The effect of bacterial diet (*ϵ*) on the probability of developing the predatory mouth form in the plastic strain (λ_P,*ϵ*_). The dotted gray line indicates the probability of developing the predatory mouth form in the non-plastic strain in both environments. The posterior distributions were generated by fitting a hierarchical bayesian model to laboratory measurements (For more information, see S1 Text and S1 Table). **(D)** Growth on *E. coli* OP50 or *Novosphingobium* sp. L76 dramatically, and differentially, affects the number of eggs laid by the adult hermaphrodites belonging to the plastic or the non-plastic strain. In our assay, the number eggs laid by a single young adult hermaphrodite, i.e., a newly matured worm, is counted during a seven-day period. This window should approximately account for 95% of the eggs produced by an adult hermaphrodite during its entire life. Given the low number of eggs laid in the last three days, data from days five, six, and seven were combined into one category. The mean values (diamond symbols) were used as fecundity values in the model. These data was previously reported in Dardiry *et al.* [40]. **(E)** The life-cycle of *P. pacificus* in our model is represented as a Markov chain where different states correspond to *P. pacificus* developmental and reproductive stages. Starvation affects transitions between states differentially, notably, the emergence of the dauer larvae, which enables dispersal. In our model, the transition rate between the young adult stage and the day-one breeding adult is differentially affect by the bacterial diet, reflecting the faster emergence of adults in *P. pacificus* observed when grown on *Novosphingobium* sp. L76 under the laboratory conditions. The starvation condition in the model is triggered when the amount resource available at time *t* is less than *βN*_*c*_(*t*), where *β* is the per capita consumption rate and *N*_*c*_(*t*) is the total number of consumers in the population at time *t*, which excludes eggs and the non-feeding dauer larvae. **(F)** In isolation and in the abundance of resources and without density-dependent mortality, our model depicts the population dynamics of the plastic and the non-plastic strains based on the laboratory measurements of fecundity on the two alternative diets. *n*_*i*_ is the count of stage *i* in a population. The dashed black lines represent the proportion of each stage for the plastic strain. The simulations started with 50 juveniles. Abbreviations: J, juvenile; YA, young adult; *RA*_i_, reproducing adult of day *i*; OA, old adult; P, plastic; NP, non-plastic.

There is no paucity of discussions on the hypothetical cost of adaptive plasticity in the ever-growing body of literature on phenotypic plasticity [6–11]. Consider a defensive phenotype *P*_d_ that confers benefit to an organism by protecting it against the predators, similar to the expression of the defensive spine in *Daphnia pulex*[12, 13]. Genotype *G*_*f*_ expresses *P*_*d*_ regardless of the presence or the absence of any predator, i.e., genotype *G*_*f*_ lacks any phenotypic plasticity with respect to this trait. In contrast, genotype *G*_*p*_ is plastic with respect to the defensive phenotype *P*_*d*_: in the presence of predators in the environment, it expresses *P*_*d*_, while in the absence of predators it expresses an alternative non-defensive phenotype (*P*_*a*_). In the absence any trade-offs, *G*_*f*_ would endure a fitness cost due to expressing a mismatched phenotype in environments that are devoid of predators, whereas *G*_*p*_ expresses the appropriate phenotype in a given environment. It has been suggested that the paucity of such plasticity-fueled master-of-all genotypes in the wild is due to the cost of plasticity, i.e., the trade-off in fitness due to the machinery required to express and maintain the plastic response [6, 7]. In our example, in an environment with predators (*E*_1_), *P*_*d*_ is the optimal phenotype with respect to predation. Since *G*_*f*_ lacks plasticity whereas *G*_*P*_ uses the presumably costly plasticity machinery to express the matching phenotype in *E*_1_, *G*_*p*_ would have a lower fitness than *G*_*f*_ in this environment: $\Delta w_{E_{1}}=w_{\left(G_{f}|E_{1}\right)}-w_{\left(G_{p}|E_{1}\right)}>0$. $\Delta w_{E_{1}}$ is equivalent to the cost of plasticity for *G*_*p*_ in environment *E*_1_. On the other hand, in an environment devoid of predators (*E*_2_), *G*_*f*_ will have a lower fitness than *G*_*p*_, since it expresses the costly defensive phenotype *P*_*d*_, while *G*_*p*_ produces the appropriate non-defensive phenotype *P*_*a*_, i.e., $\Delta w_{E_{2}}=w_{\left(G_{f}|E_{2}\right)}-w_{\left(G_{p}|E_{2}\right)}<0$. It should be noted that $\Delta w_{E_{2}}<0$ is valid only if the cost of simply possessing a plastic machinery by *G*_*p*_, i.e., when it is not induced to express *P*_*d*_, does not exceed the cost of phenotypic mismatch between *P*_*d*_ expressed by *G*_*f*_ and environment 2 (Fig 1A).

While the concept of cost of plasticity is the logical extension of the general discussion on limits and constrains of evolution (e.g., see [14]), they pose non-trivial practical obstacles. Firstly, these costs can only be investigated if and when the phenotypically plastic trait proves to be adaptive, since, as pointed out by Bradshaw, “the concept of plasticity does not also have any implications concerning the adaptive value of the changes occurring […]” [5]. However, the simple act of assigning adaptive value to a trait, while almost trivial in theory, can be challenging in practice [15–17]. Secondly, the measurement of a cost associated with phenotypic plasticity as such is inevitably confounded with other factors, including the cost of expressing a maladptive phenotype in an environment (for an in-depth discussion, see [18]). To empirically study any evolutionary tradeoff is inherently challenging [19–21], and the cost of plasticity is no exception in this respect.

Despite such obstacles, many attempts to measure the cost of adaptive plasticity in nature have been made (e.g., [22–24]). The general design of such studies involves finding a plastic trait that can be plausibly characterized as adaptive with regard to a given environmental condition, and measuring a component of fitness, e.g., fecundity, size, etc., across two or more conditions, one being the condition to which the plastic response is adapted. While such studies should, in principle, demonstrate the cost of plasticity, they have provided mixed evidence; a meta-analysis of 27 studies of the cost of adaptive plasticity concluded that the costs measured in these studies are quite infinitesimal, if present at all [25]. Surprisingly, while *Daphnia* is sometimes used as a visual aide to illustrate the cost of adaptive plasticity (e.g., [26]), the induction of the defensive spine in *Daphnia pulex*, in response to a predator (*Chaoborus americanus*), was shown to have negligible cost in spite of a forgiving statistical approach [27].

It should be pointed out that the relationship between the average fitness of individuals of the plastic genotype *i* in environment *j*, usually measured via the number of offspring produced during a time period, and the cost of plasticity is anything but elementary. The cost of plasticity was formulated with respect to the plausibility of the evolution of adaptive phenotypic plasticity [7], thus, the value of fitness relevant to the cost of plasticity should be measured across many generations. However, the cost of plasticity is usually measured within a single generation across conditions (e.g., [23, 24]). Such snapshots could mislead us about the realized cost of plasticity in nature, given that factors such as environmental fluctuations, frequency-dependent selection, or different bet-hedging strategies can result in evolutionary scenarios where selection does not optimize the population growth rate [28]. In this respect, in the absence of experimental evolution or long-term field data, combining experimental measurements with mathematical models to predict the effect of the cost of plasticity over generations can provide insights about the ecological relevance of the cost of plasticity.

The hermaphroditic nematode *Pristionchus pacificus* provides an interesting case study to better illustrate the complications of inferring the realized cost of plasticity from laboratory measurements of fecundity. *P. pacificus* is a prominent model system to study phenotypic plasticity with well-established genetic, molecular, and experimental tools [29–34]. Importantly, the hermaphroditic mode of reproduction of this nematode results in isogenic cultures, in which all individuals are genetically identical. Nonetheless, the mouth form of *P. pacificus* can assume two alternative states: a wide eurystomatous (predatory) form with two teeth, which enables the nematode to prey upon other nematodes, and a narrow bacterivorous stenostomatous (non-predatory) form with a single tooth (Fig 1B). The state of the mouth form can be influenced by a variety of stimuli, including temperature, culture methods, pheromones, and bacterial diet [35–38]. In addition to change in mouth form due to environmental cues, different wild isolates of *P. pacificus* exhibit a range of mouth-form ratios under laboratory condition [39].

To illustrate how environment affects the mouth form polyphenism, we focus on two isogenic *P. pacificus* strains: RSC017, the plastic (P) strain, and RS5405, the non-plastic (NP) strain. The mouth-form of individual *i* of strain *j* in environment *ϵ*, *y*_*i*,*j*_, can be modelled such that *y*_i,j_ ∼ *Bernoulli*(λ_j,*ϵ*_). Thus, the polyphenism in *P. pacificus* can be characterized by estimating λ_*j*,*ϵ*_—i.e., the probability of developing the predatory mouth form by an adult of strain *j* in the environment *ϵ*—across environmental conditions. Previously [40], we reported how the probability of developing the predatory mouth form of the plastic strain increases drastically when grown on *Novosphingobium* sp. L76 (the inducing diet) relative to *E. coli* OP50 (the non-inducing diet) (Fig 1C). In contrast, the non-plastic strain is an obligate predator across both conditions. Both bacteria strongly differ in their nutritional value and were shown to influence various life history traits of the nematode [38, 40]. These observations are likely of ecological relevance because the *Novosphingobium* sp. L76 strain was originally isolated from a *Pristionchus* environment [38, 41]. Indeed, when we measure the number of eggs laid by adult hermaphrodites of the plastic and the non-plastic strains during a 7-day period under the inducing and the non-inducing conditions, *Novosphingobium* sp. L76 and *E. coli* OP50, respectively, a pattern consistent with the expected cost of plasticity and the cost of phenotypic mismatch between a fixed genotype and an environment emerges: the fecundity of the plastic strain decreases on the inducing diet relative to its fecundity on the non-inducing diet, while the opposite pattern is observed for the non-plastic strain (Fig 1D).

However, there are several reasons to assume that the relation between the experimental measurements of fecundity, measured at the individual level, does not provide a comprehensive picture as to how the cost of plasticity would affect comeptition between a plastic and non-plastic strains of *P. pacificus*. The mouth-form polyphenism in *P. pacificus* results in stage-specific intraguild predation, a phenomenon usually referred to as life-history intra-guild predation (LHIGP) [42], where adults with a predatory mouth form prey upon the juveniles of other strains. Such intra-guild predation can drastically change the competitive outcomes [43, 44]. In addition, the life cycle of *P. pacificus* in nature further complicates the ecological consequences of the cost of plasticity and the cost of phenotypic mismatch between a fixed genotype and a given environment: *P. pacificus* and its relatives are soil nematodes that are most reliably found in association with scarab beetles [45, 46]. These nematodes stay in the arrested dauer larval stage (an alternative larval stage) as long as the adult beetle is alive and flourish on the beetle cadaver in the soil once the beetle has died [47, 48]. The importance of dispersal and colonization, coupled with LHIGP, in *P. pacificus* suggests that a simple, possibly linear, relationship between the individual-level laboratory measurements of fecundity in the inducing and the non-inducing conditions, is not a given.

Additionally, understanding how spatial and/or temporal heterogeneity of the environment would affect the costs of phenotype and plasticity is crucial, since the evolution of phenotypic plasticity is often discussed in the context of spatial or temporal environmental fluctuations, situations where a fixed strategy does not guarantee evolutionary success [11, 49–52]. Specifically, the predatory nature of the plastic phenotype in *P. pacificus* raises an interesting question: if we include the induced phenotype, does the predation of juvenile and dauer larval stages of the non-plastic strain by the plastic strain offset the cost of plasticity and, to what extent the purported costs of plasticity and phenotypic mismatch affect the ecological consequences of mouth-form plasticity? Taken together, the confluence of the aforementioned factors that could modulate the cost of plasticity across time and space could also shed some light on the role of phenotypic plasticity in ecological coexistence (reviewed in [53]). Investigating this aspect of phenotypic plasticity, which has recently gained much-deserved attention (e.g., see [54]), is fundamental to integrating phenotypic plasticity within the broader ecological context.

Here we present a stage-structured metapopulation model consisting of *m*^2^ populations arranged on an *m*×*m* lattice. We incorporate experimentally-estimated parameters for developmental speed, fecundity, and mouth-form plasticity of the plastic and the non-plastic *P. pacificus* strains into the metapopulation model. Using this model, we attempt to answer the following questions:

- Can spatial or temporal heterogeneity, or both, with respect to the bacterial resource alleviate the cost of plasticity in the plastic strain?
- How much does LHIGP affect the costs of plasticity and phenotype in *P. pacificus*?
- How does dispersal affect the possibility of coexistence of the plastic strain in the presence of non-plastic strain?

## Materials and methods

To simulate the population dynamics of the interaction between the plastic and the non-plastic strains of *P. pacificus*, we use a modified version of a stage-structured matrix population model [40]. In this model, we envision the life cycle of *P. pacificus* as an absorbing finite-state Markov chain [55, 56] (Fig 1E). The life cycle consists of egg (E), juvenile (J), dauer larvae (d), young adult (YA), reproducing adult (RA), and old adult stages (OA). As noted before, growth on *E. coli* OP50 or *Novosphingobium* sp. L76 changes the number of predatory adults in the plastic strain by affecting the probability of developing the predatory mouth form at the individual level. In addition, these two diets dramatically affect the total number of eggs produced by adult hermaphrodites of the plastic and the non-plastic strain, as well as the developmental speed of the worms, notably in the YA to RA transition [40].

The effect of diet on fecundity is incorporated in our model via the fertility matrix **F**_*i*_(*ϵ*), where *ϵ* indicates the type of diet available, i.e., being grown on *E. coli* OP50, *Novosphingobium* sp. L76. Given that our experimental measurement of fecundity encompasses 5 days (Fig 1D), five reproducing adult stages (RA_1_ to RA_5_) were included in the markov chain representing the life-cycle of *P. pacificus*. The entry for the reproducing adult of strain *i* of day *j* in the fertility matrix is defined as $\phi_{i,j}\left(∊\right)={\bar{z}}_{i,j}\left(∊\right)\gamma_{j\to j+1}\left(∊\right)$, where ${\bar{z}}_{i,j}\left(∊\right)$ is the mean number of eggs laid by a *j* day old adult of strain *i* grown on the diet *ϵ*, and *γ*_*j* → *j*+ 1_(*ϵ*) is the transition probability from the current to the next developmental state in strain *i* given *ϵ*. The fertility matrix **F**_*i*_(*ϵ*) is

$$\begin{array}{c} F_{i}\left(\epsilon \right)=(\begin{array}{cccccccc} 0 & \cdots & \phi_{i,1}\left(\epsilon \right) & \phi_{i,2}\left(\epsilon \right) & \phi_{i,3}\left(\epsilon \right) & \phi_{i,4}\left(\epsilon \right) & \phi_{i,5}\left(\epsilon \right) & 0\\ 0 & \cdots & 0 & 0 & 0 & 0 & 0 & 0\\ \vdots & \cdots & \vdots & \vdots & \vdots & \vdots & \vdots & \vdots \\ 0 & \cdots & 0 & 0 & 0 & 0 & 0 & 0 \end{array})\;. \end{array}$$ (1)

The formation of dauer larvae is a fascinating feature of the *P. pacificus* life cycle, enabling the nematode to transition to this alternative developmental stage in response to harsh conditions. This non-feeding and resilient stage, which can survive up to 50 weeks under laboratory conditions, disperses in the environment, and resumes the normal development once it encounters favorable conditions [48, 57, 57–59]. To include the effect of this stage on the competition between the plastic and the non-plastic strains of *P. pacificus*, a simple linear resource consumption model was included. Given an amount of available resource, either *E. coli* OP50 or *Novosphingobium* sp. L76 at time *t*, $R_{t}$, the amount of available resource at the next step will be:

$$\begin{array}{c} R_{t+1}=R_{t}-\beta N_{c}\;, \end{array}$$ (2)

where *β* is the consumption rate and *N*_*c*_ is number of consumers in the population, which excludes eggs and dauer larvae. Starvation is assumed when $R_{t}<R_{\text{min}}$, where $R_{\text{min}}=\beta N_{c}$.

The life cycle of strain *i* in resource state *ϵ* is determined by its transition matrix **U**_*i*_(*ϵ*):

$$\begin{array}{c} U_{i}\left(\epsilon \right)=\left(\begin{array}{llllllll} \text{E} & \text{J} & \text{d} & \text{YA} & {\text{RA}}_{\text{1}} & \ldots & {\text{RA}}_{5} & \text{OA}\\ \sigma_{1}(N_{c})(1-\gamma_{21}(\epsilon )) & 0 & 0 & 0 & 0 & \ldots & 0 & 0\\ \sigma_{1}(N_{c})\gamma_{21}(\epsilon ) & \sigma_{2}(N_{c})(1-\gamma_{32}(\epsilon ))(1-\gamma_{42}(\epsilon )) & 0 & 0 & 0 & \ldots & 0 & 0\\ 0 & \sigma_{2}(N_{c})\gamma_{32}(\epsilon ) & \sigma_{3}(1-\gamma_{43}(\epsilon )) & 0 & 0 & \ldots & 0 & 0\\ 0 & \sigma_{2}(N_{c})\gamma_{42}(\epsilon ) & \sigma_{3}\gamma_{43}(\epsilon ) & \sigma_{4}(N_{c})(1-\gamma_{54}(\epsilon )) & 0 & \ldots & 0 & 0\\ 0 & 0 & 0 & \sigma_{4}(N_{c})\gamma_{54}(\epsilon ) & \sigma_{5}(N_{c})(1-\gamma_{65}(\epsilon )) & \ldots & 0 & 0\\ \vdots & \vdots & \vdots & \vdots & \vdots & \vdots & \vdots & \vdots \\ 0 & 0 & 0 & 0 & 0 & \ldots & \sigma_{9}(N_{c})(1-\gamma_{109}(\epsilon ))\; & 0\\ 0 & 0 & 0 & 0 & 0 & \ldots & \sigma_{9}(N_{c})\gamma_{109}(\epsilon ) & \sigma_{10}(\epsilon ) \end{array}\right) \end{array}.$$

The transition probabilities are affected by the diet *ϵ* and starvation (Fig 1E and S1 Fig). The survival probability of all stages, except for the dauer larvae, is influenced by population density: $\sigma \left(N_{c}\right)=e^{-\psi N_{c}}$, where *ψ* is a fixed parameter determining the intensity of the density-dependent mortality (S2 Fig). Given its resilience and longevity, for dauer larvae *σ*_3_ = 1.

The population is represented by a vector

$$n=\left(\frac{\begin{array}{c} n^{i1}\\ \vdots \\ n^{i10} \end{array}}{\begin{array}{c} n^{j1}\\ \vdots \\ n^{j10} \end{array}}\right)\;,$$ (3)

where *n*^*lm*^ represent the number of individuals that belong to stage *m* of the strain *l*. The composition of the population in the next step, without considering predation and dispersal, will be

$$\begin{array}{c} n_{t+1}=A\left(\epsilon \right)n_{t}\;, \end{array}$$ (4)

where

$$\begin{array}{c} A\left(\epsilon \right)=(\begin{array}{cc} U_{i}\left(\epsilon \right)+F_{i}\left(\epsilon \right) & \\ & U_{j}\left(\epsilon \right)+F_{j}\left(\epsilon \right) \end{array})\;. \end{array}$$ (5)

The effect of predation of strain *j* on strain *i* at each step is included as a type II predation (S3 Fig). The number of dauer larvae of strain *i* killed at time *t* ($\delta n_{t}^{i,d}$) is:

$$\begin{array}{c} \delta n_{t}^{i,d}=\frac{an_{t}^{i,d}}{1+ahn_{t}^{i,d}}N_{t}^{j,p}\;, \end{array}$$ (6)

where $N_{t}^{j,p}$ is the number of predators of strain *j* in the population at time *t*, *a* denotes the attack rate, and *h* represents the handling time. The number of juveniles of strain *i* killed at time *t* is calculated using Eq 6. $N_{t}^{j,p}$ equals the expected number of young and breeding adults of strain *j* with the predatory mouth form, determined by the probability of developing the predatory mouth from in a given environment for strain *j* (λ_*j*,*ϵ*_).

In nature, upon the depletion of bacteria on the beetle carcass, *P. pacificus* dauer larvae are generated and rapidly disperse in the surrounding soil [48]. To simulate the dispersal of dauer larvae over the metapopulation, in each step, proportion *r* of the dauer larvae of strain *i* in subpopulation *s*_*x*,*y*_ disperse to each of its valid neighboring subpopulations in a von Neumann neighborhood: *s*_*x* + 1, *y*_, *s*_*x*−1, *y*_, *s*_*x*,*y*+ 1_, and *s*_*x*,*y*−1_.

The expected composition of the metapopulation at time *t* + 1 is calculated in three steps:

1. For each of the *m*^2^ subpopulations in the metapopulation, the expected population composition without predation at *t* + 1 is calculated using Eq 4.
2. The effect of predation on the juvenile and dauer larvae in each subpopulation is calculated using Eq 6.
3. The dauer larvae disperse across the metapopulation from any given subpopulation to its neighbors.

## Software

The software used to run all simulation was written in Python 3.11.5 with NumPy 1.25.2 [60]. Bayesian analysis was conducted using PyMC version 5.9.1 [61].

## Results

### Costs of plasticity and phenotype in a mixed population with finite resource

We used experimentally-derived life history parameters of two wild isolates of *P. pacificus* to study the cost of plasticity [40]. Before simulating competition between the plastic and the non-plastic strains, we simulated the population dynamics of each strain individually without resource limitation and density-dependent mortality (Fig 1F). In isolation, the dynamics of the plastic and the non-plastic strains is consistent with both the cost of phenotypic mismatch between a fixed genotype and a given environment, i.e., the lower growth rate of the non-plastic strain in the non-inducing environment (*E. coli* OP50) relative to the plastic strain, and the cost of plasticity, i.e., the lower relative growth rate of the plastic strain in the inducing environment (Fig 1D). However, these results by definition do not consider the crucial competitive ramifications of the mouth-form plasticity in *P. pacificus*, since the induced phenotype in the plastic strain exhibits an increase in the proportion of the predatory mouth from 0.11 on *E. coli* OP50 to 0.83 on *Novosphingobium* sp. L76 (Fig 1C). Following the schematic representation introduced in the introduction (Fig 1A), we use $\Delta w_{E_{1}}$ to denote the cost of plasticity, i.e., the lower fecundity of the plastic strain relative to the non-plastic strain in *Novosphingobium* sp. L76, and $\Delta w_{E_{2}}$ to refer to the relative cost of phenotypic mismatch between the predatory phenotype of the non-plastic strain and *E. coli* OP50, i.e., its lower fecundity relative to the plastic strain on this diet.

**Fig 2:**
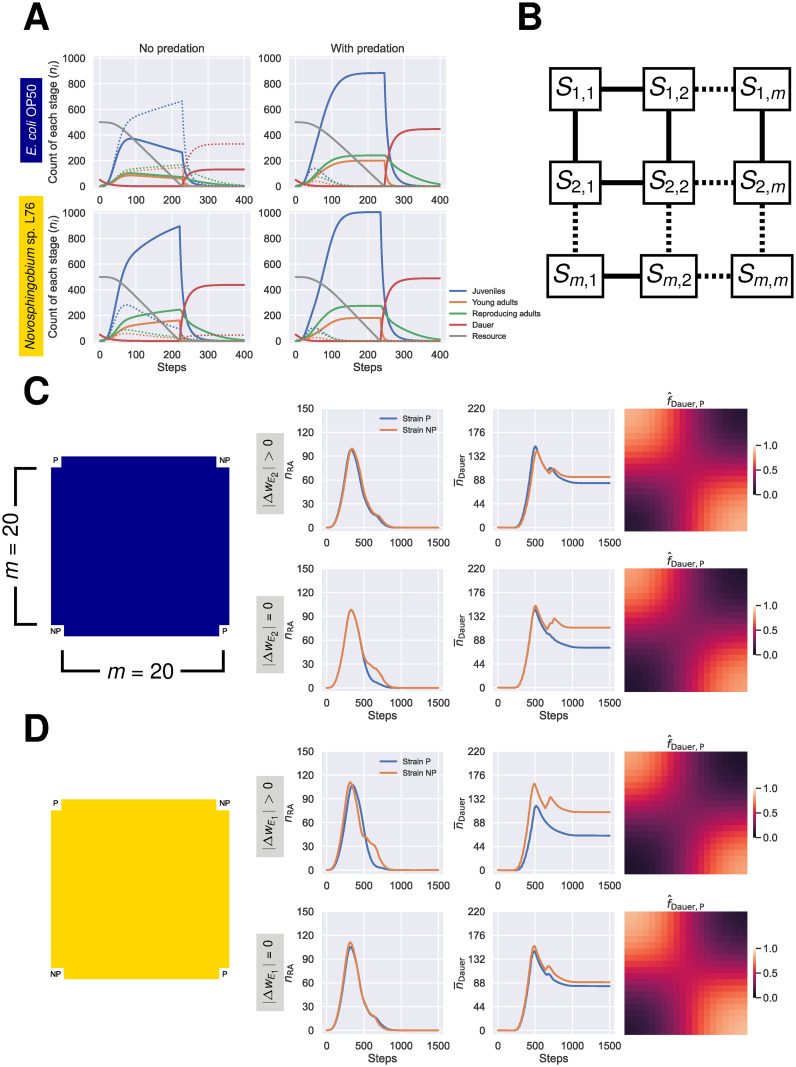
Cost of plasticity in well-mixed versus spatially-structured populations. **(A)** The expected outcome of competition between the non-plastic (solid lines) and the plastic strains (dotted lines) in a population with limited resource and density-dependent mortality. In the absence of predation, the plastic strain would out-compete the non-plastic strains on *E. coli* OP50 given its higher per-generation growth rate. If predation is included, the non-plastic strain is always eliminated by the plastic strain, regardless of the bacterial diet. **(B)** The meta-population in our model consists of *m* × *m* subpopulations (*S*_1,1_ to *S*_*m*,*m*_), on a lattice. The dispersal in the metapopulation is simulated by movement of the dauer larvae from each population to its neighboring populations. **(C)** The effect of the relative cost of phenotypic mismatch between the phenotype of the fixed genotype—i.e., its mouth form—and environment 2 ($\Delta w_{E_{2}}$) on the competition between the plastic and the non-plastic strains. The simulation started on a 20 × 20 lattice, with four populations seeded with 50 dauer larvae of the plastic (P), on locations *S*_1,1_ and *S*_*m*,*m*_, or the non-plastic (NP) strains, on locations *S*_1,*m*_ and *S*_*m*,1_. Each population on the lattice started with *R*_0_ = 500 quantity of *E. coli* OP50. If $\Delta w_{E_{2}}=0$, the fecundity of the non-plastic strain on *E. coli* OP50 (the non-inducing environment) is identical with its fecundity on *Novosphingobium* sp. L76 (the inducing environment). ${\bar{n}}_{i}$ is the mean number of stage *i* per population. ${\hat{f}}_{\text{Dauer},P}$ was calculated by dividing the final number of dauer larvae of the plastic strain by the total number of dauer larvae in a given population. **(D)** The effect of the cost of plasticity ($\Delta w_{E_{1}}$) on the competition between the plastic and the non-plastic strains. Each population on the lattice started with *R*_0_ = 500 quantity of *Novosphingobium* sp. L76. If $\Delta w_{E_{1}}=0$, the fecundity of the plastic strain on *Novosphingobium* sp. L76 is identical with its fecundity on *E. coli* OP50. Abbreviations: RA; reproducing adults. Parameters: consumption rate = 0.002, type II predation *a* = 0.2 and *h* = 0.15, dispersal parameter *r* = 0.01.

We next simulated the dynamics of the two strains in a well-mixed population that includes a finite resource, with and without predation (Fig 2A). In these and the following simulations in this section, the consumer stages, i.e., all the stages except for the egg and the dauer larvae, suffer from a density-dependent mortality, as described in Materials and methods. In the absence of predation, the plastic strain reaches a higher abundance in all stages compared to the non-plastic strain on *E. coli* OP50, given its higher relative growth rate, while it fares worse on *Novosphingobium* sp. L76. Once we include predation, the non-plastic strain always competitively excludes the plastic strain regardless of the diet since it is 100% predatory across the environmental gradient while a portion of the juvenile larvae of the plastic strain develop into non-predatory even on the inducing diet (*Novosphingobium* sp. L76). Thus, the magnitude of the induced phenotype in the plastic strain is not enough to offset the lower relative growth rate of this strain on *Novosphingobium* sp. L76 (Fig 2A). However, the well-mixed condition is an extreme case, and in nature one would expect heterogeneity both in space and time. To capture these aspects, we constructed a metapopulation model (Fig 2B). Dauer larvae disperse between neighboring populations with parameter *r*.

### Costs of plasticity and phenotypic mismatch hamper competitive capability in a homogeneous metapopulation

In the simplest scenario, localities are assigned identical diet, that is the fecundity and the probability of developing the predatory mouth form for each strain does not change across the lattice. The plastic and the non-plastic strain would compete over the resources distributed over the lattice by dispersing to neighboring locations, via the dauer stage, colonizing that location, and generating more dauer larvae to disperse and compete over the ever-diminishing resources. On *E. coli* OP50, the higher growth rate of the plastic strain relative to the non-plastic strain ($\Delta w_{E_{2}}$) almost equalizes the count of dauer larvae the two strains, with the final count of dauer larvae of the non-plastic strain over the lattice being only slightly higher than that of the plastic strain. Had the fecundity of the non-plastic strain been free of the cost of phenotypic mismatch ($\Delta w_{E_{2}}=0$), that is, had the fecundity of this strain remained as high in the non-inducing environment as it is in the inducing one, the non-plastic would have fared much better in this competition (Fig 2C). The same scenario, when applied to a lattice seeded with *Novosphingobium* sp. L76, demonstrates how the cost of plasticity ($\Delta w_{E_{1}}$) hampers the competitive capability of the plastic strain (Fig 2D). In this condition, the lower fecundity, in spite of the higher expression of the predatory mouth in the plastic strain, results in lower number of dauer larvae of this strain relative to that of the non-plastic strain at the end of the competition. Without the reduction in the fecundity of the plastic strain on the inducing environment ($\Delta w_{E_{1}}=0$), the plastic strain would have performed as well as the non-plastic strain. These results can be attributed to LHIGP (S4 Fig).

### Initial resource heterogeneity can alleviate the cost of plasticity

**Fig 3:**
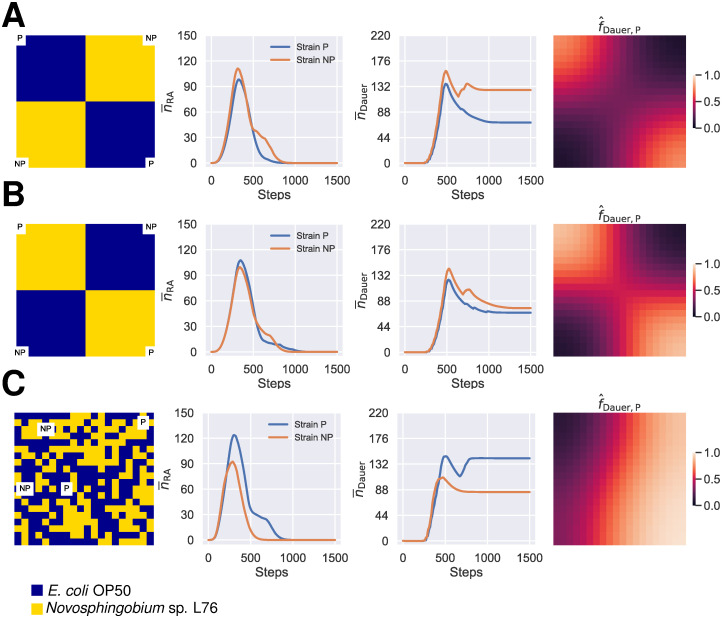
The effect of initial resource heterogeneity on the competition between the plastic and the non-plastic strains. **(A)** The metapopulation was divided into quadrants and each quadrant was assigned an alternative resource at the start of the simulation, such that subpopulations seeded with plastic dauer larvae (*S*_1,1_ and *S*_*m*,*m*_) are within *E. coli* OP50 quadrants and subpopulations seeded with non-plastic dauer larvae (*S*_1,*m*_ and *S*_*m*,1_) within *Novosphingobium* sp. L76 quadrants. **(B)** An alternative arrangement of the quadrants where used as the inital composition of the two alternative resources on the lattice, where subpopulations seeded with plastic dauer larvae are within *E. coli* OP50 quadrants and subpopulations seeded with non-plastic dauer larvae within *Novosphingobium* sp. L76 quadrants. **(C)** The simulation where both the initial location of the populations seeded by the dauer larvae and the assignment of resource type to each population were randomized. ${\bar{n}}_{i}$ is the mean number of stage *i* per population. ${\hat{f}}_{\text{Dauer},P}$ was calculated by dividing the final number of dauer larvae of the plastic strain by the total number of dauer larvae in a given population. Abbreviations: RA; reproducing adults.

In nature one would expect a heterogeneous distribution of resources, both with regard to amount and type. To explore the effect of non-homogeneous distribution of resource type in the environment, we simulated the population dynamics over metapopulations with two arbitrary resource distribution patterns: in each pattern, the metapopulation was divided into four quadrants and either *E. coli* OP50 or *Novosphingobium* sp. L76 was assigned to each quadrant (Fig 3A and 3B). The expectation is that, given the effect of each diet on developmental speed, specifically (YA → RA_1_), and fecundity of the two strains, these heterogeneities will influence the realized costs of plasticity and phenotypic mismatch for the non-plastic strain, such that the competitive outcomes over the metapopulation will be altered. The first simple pattern, where the two populations seeded with the dauer larvae of the plastic strain are located on the two quadrants that have *E. coli* OP50 as their resource, the non-plastic strain outcompetes the plastic strains and leaves more dauer larvae behind in the metapopulation at the end of the competition (Fig 3A). However, the alternative pattern, where the two populations seeded with the dauer larvae of the plastic strain are located on the two quadrants that have *Novosphingobium* sp. L76 as their resource, the two strains perform equally well (Fig 3B). These resutls are also strongly influenced by LHIGP (S5 Fig). Since such geometrically strict patterns of resource distribution are not expected in nature, we also simulated metapopulations where the type of resource, *E. coli* OP50 or *Novosphingobium* sp. L76, was assigned at random to each population at the start of the simulation. In addition, we allowed the location of the starting populations seeded with the dauer larvae of each strain to be randomly selected (Fig 3C). Such heterogeneities in resource distribution and the initial location of the starting populations results in scenarios where the plastic strain outcompetes the non-plastic strain (Fig 3C). However, under these conditions, the plastic strain is not always favored (S6 Fig).

### Spatial and temporal heterogeneities alter the cost of plasticity

There are two limitations to the previous exploration of the effect of spatial heterogeneity in resource distribution: firstly, the population dynamics in that experimental design reflect a single “boom and bust” phase, where a subpopulation is colonized by dauer larvae, resources are consumed by the developing worms and dauer larvae are again generated upon the depletion of the resource, until no resource is available in the metapopulation. Laboratory data suggest that *P. pacificus* follows many boom and bust rounds in nature, where successive growth periods of bacteria results in cycles of population growth and waves of dauer dispersion in the same locality [48]. Secondly, the single boom and bust phase does not allow us to investigate the possibility of long-term coexistence or exclusion in a metapopulation that consists of both the plastic and the non-plastic strains.

**Fig 4:**
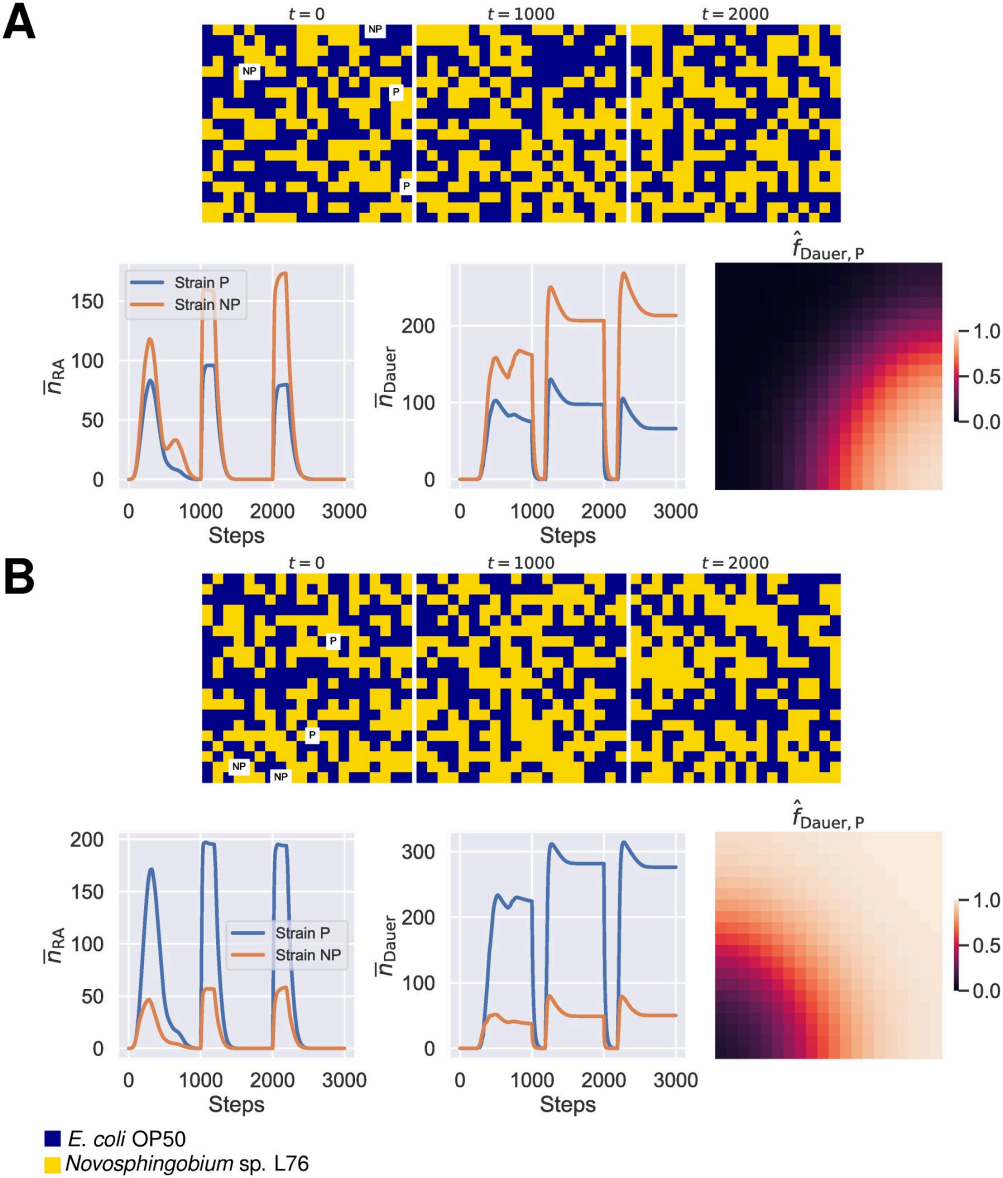
The effect of temporal and spatial heterogeneity on the competition between the plastic and the non-plastic strains. To simulate competition between the plastic and the non-plastic strains in a facsimile of the *P. pacificus* boom-and-bust cycles, every 1000 steps we replenished the resources in the metapopulation and randomly reassigned resource types to each population. The initial location of the four populations seeded with the plastic or non-plastic dauer larvae at the start of simulations were also randomized. Such conditions can result in scenarios where the non-plastic **(A)** or the plastic strain **(B)** dominates the metapopulation. ${\bar{n}}_{i}$ is the mean number of stage *i* per population. ${\hat{f}}_{\text{Dauer},P}$ was calculated by dividing the final number of dauer larvae of the plastic strain by the total number of dauer larvae in a given population. Abbreviations: RA; reproducing adults.

To address these limitations, in addition to the initial spatial heterogeneity in resource distribution and the location of the starting populations seeded with dauer larvae, we replenished resource at fixed intervals during simulations, to represent a facsimile of the expected natural boom and bust periods in the *P. pacificus* life cycle (Fig 4). Heterogeneities introduced in such cycles can result in all-or-nothing outcomes. However, in some cases, they allow for transient coexistence of both the plastic and the non-plastic strains to be found in the metapopulation. The plastic strain can become the dominant strain in the metapopulation under these conditions (Fig 4 and S7 Fig).

## Discussion

Phenotypic plasticity is often discussed in the context of a changing environment [11, 62]. The recent and growing body of literature on the role of plasticity in adapting to the ever-changing anthropogenic conditions of the biosphere has brought this aspect of plasticity in sharper relief [52, 63–67]. However, the presumed benefits of plasticity, as the source of “jack-of-all-trades” phenotypes, is undermined when the cost of plasticity is taken into account. This concept, most comprehensively articulated by DeWitt *et al.* in their much-cited theoretical contribution on this topic [7], suggests that a variety of features of any phenotypically-plastic system, e.g., production, information acquisition, and maintenance, would make a plastic system inherently costly compared to a non-plastic variant. As pointed out before, the purported sources of cost of plasticity combine environment-dependent and environment-independent factors [68].

There exists a large body of literature on the conditions that favor the evolution of phenotypic plasticity, where the general patterns of spatial and temporal variations that would result in the evolution or maintenance of plastic traits are delineated (e.g., [49, 50, 67, 69]). But such models, by necessity, provide broad predictions, with results that are entirely dependent on the adaptive nature of plasticity and its associated cost. For example, a recent contribution by Scheiner *et al.* [67] on phenotypic plasticity and climate change, ends with a valuable discussion on how their assumptions about the cost and adaptive state of the plastic trait provided outcomes quite different from other theoretical treatments of the subject, such as Nunney’s [70].

**Fig 5:**
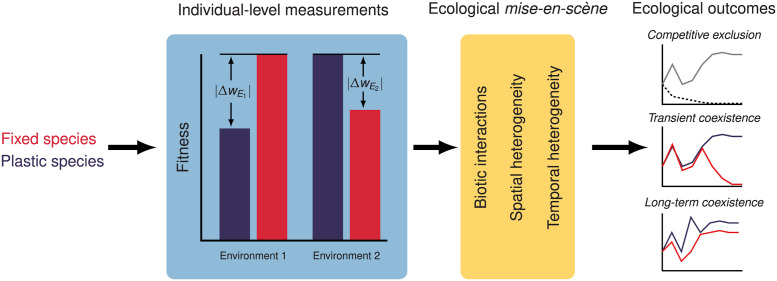
The roles of costs of plasticity and phenotypic mismatch in determining the outcome of ecological competition. The usual approach to measure the cost of phenotype ($\Delta w_{E_{1}}$) and the relative cost of phenotypic mismatch between the phenotype of a fixed genotype and its environment ($\Delta w_{E_{2}}$) involves the comparative individual-level measurements of one or several life-history traits, as a proxy for fitness. In our study, while the ease at which we can experimentally measure traits in *P. pacificus* provides a relatively accurate readouts, such costs, once filtered through the lens of ecological factors (*ecological mise-en-scène*) can result in a wide range of ecological outcomes.

The quantification of the cost of plasticity is crucial to understand the evolutionary causes of phenotypic plasticity [71]. While theoretical works on the cost of plasticity, from the pioneering contribution by Van Tienderen [72] to the more recent contributions, e.g., [50, 73], have explored the conditions under which phenotypic plasticity might persist, our attempt in this contribution is to illustrate how costs of phenotype and plasticity for a given trait, measured in isolation, and in multiple environmental conditions might not provide enough information to predict the realized costs burdened by the same organisms in nature. Conceptually, we want to understand how individual-level measurements of fitness proxies might be related to how competition unfolds in the ecological theater [74] (Fig 5). Only modeling approaches as the one described in this study can provide the specificity required to move beyond a generic prediction to a deeper understanding of the potential ecological ramifications of phenotypic plasticity in particular case studies.

As shown in a previous contribution [40], under the laboratory conditions, the costs of plasticity and phenotypic mismatch for the non-plastic strain, inferred based on the experimental measurements of the vital rates of two strains of *P. pacificus* across two conditions, were considerable and consistent with conceptual expectation of espoused by DeWitt *et al.* [7] and related works. However, the boom-bust population dynamics of *P. pacificus* in nature should make the ecological manifestation of these costs more complex. This expectation stems from the observation that, even without considering phenotypic plasticity, boom-bust dynamics can greatly affect the composition of ecological communities [75].

The coexistence of species remains an elusive problem in ecology [76]. There has been attempts to provide a satisfying answer to this problem, including mechanistic models such as the modern coexistence theory [77], where mechanisms that equalize the fitness of the competitors and mechanisms that increase negative intraspecific interactions relative to negative interspecific ones promote coexistence. It has also been suggested that species (and presumably strains) that are alike can also lump together from competitive interactions [78]. However, how phenotypic plasticity would affect the possibility of coexistence is still an open question (reviewed in [53]). Our attempt in this contribution is a step in providing some insight concerning how phenotypic plasticity can affect competition.

It should be noted that the results presented here, while providing a species-specific prediction with regard to the cost of plasticity and its ecological consequence, are still in parts rooted on assumptions that have yet to be thoroughly investigated in the lab. Firstly, our knowledge on the dispersal of dauer larvae in the wild is still nascent, with many aspects that remain elusive [48, 59]. Do dauer larvae of the plastic strains of *P. pacificus* disperse at different rates compared to the non-plastic ones? Indeed, it has been suggested that the evolution of higher dispersal rate may be favored when trait plasticity is high [79]. How does the alternative transition from dauer larval stage to adulthood, in comparison with the default path to adulthood through juvenile larvae, affect the expression of the predatory mouth form? Any of these aspects will greatly influence the ecological dynamics. Secondly, the predation dynamics in *P.pacificus*, in spite of ongoing research on the topic [80], is not fully characterized, and it is not clear how to best describe the functional response in this nematode. Similarly, to what extent the predatory behavior is dynamically affected by the presence of resource and the composition of the population is currently unknown. These limitations highlight the Herculean task facing any attempt to integrate our knowledge gained from natural history perspective and experimental manipulations, in this case our understanding of nematode propagation and dispersal in a soil beetle ecosystem and their predatory behavior, with ecological, and ultimately evolutionary, predictions. Such attempt at integration of knowledge would most likely result in a dim reflection the processes and interaction of interest, which, while frustrating, will ultimately provide the most comprehensive approach to answer a biological question that spans across organizational and temporal scales.

In spite the aforementioned limitations, the current contribution, taken together with our previous attempt to integrate experimental and modeling approaches to illustrate the costs of plasticity and phenotypic mismatch in *P. pacificus* [40], provides an important starting point in our attempt to provide a comprehensive species-specific portrait of phenotypic plasticity and its importance to supplement the extensive body of literature on phenotypic plasticity as a ubiquitous phenomenon.

## Supporting information

S1 Text The details of the Bayesian model used for estimating λ_P,*ϵ*_ in Fig 1C. (PDF)

S1 Table Laboratory data on the mouth-form plasticity of *P. pacificus* RSC017 across two bacterial conditions. (PDF)

S1 Fig The effect of the diets and starvation on the transition probabilities. *Novosphingobium* sp. L76 deferentially affects the transition between YA and day-one reproducing adult (RA_1_) in the plastic (P) and the non-plastic strains (NP), reflecting our experimental observations. The transition probabilities are chosen such that the occupancy time in the Markov chain, calculated using the fundamental matrix (**N** = (**I** − **U**)^−1^), approximately corresponds to the life-cycle of *P. pacificus* in hours, e.g., transition probability 0.0415 results in an occupancy time of ≈ 24 steps.
(EPS)

S2 Fig Density-dependent mortality. For all stages except the dauer larvae, survival probability changes as function of the number of consumers in the population, *N*_*c*_. For all results, *ψ* = 6 × 10^−5^.
(EPS)

S3 Fig Type II predation model. For the predation, attack rate (*a*) and handling time (*h*) are set at 0.2 and 0.15, respectively.
(EPS)

S4 Fig The effect of predation on metapopulation dynamics with homogeneous resource distribution. The results of the model with conditions identical to Fig 2C and 2D without predation.
(EPS)

S5 Fig The effect of predation on metapopulation dynamics with simple spatial resource distribution patterns. The results of the model with conditions identical to Fig 3A and 3B without predation.
(EPS)

S6 Fig Random initial distribution of resource does not always favor the plastic strain. Simulations with identical conditions to Fig 3C. The random initial distribution of resources over the metapopulation and random assignment of the populations seeded by the dauer larvae of either of the two strains can result in scenarios where the plastic strain loses.
(EPS)

S7 Fig The effect of temporal and spatial heterogeneity on the competition between the plastic and the non-plastic strains. Addition examples of simulations as described in Fig 4.
(EPS)
