# Supplementary material for: Spatial and temporal heterogeneity alter the cost of plasticity in Pristionchus pacificus
Source: PLoS Comput Biol. 2024 Jan 30;20(1):e1011823. doi: 10.1371/journal.pcbi.1011823 (PMC10857712; doi:10.1371/journal.pcbi.1011823)
Supplement: S1 Text — (PDF) [file pcbi.1011823.s001.pdf]

S1 Text. The details of the Bayesian model used for estimating $\lambda_{\mathrm{P},\epsilon}$

To estimate the probability of the expression of the predatory (Eu) mouth form (λ) of the plastic strain in environment ϵ, we used the experimental data (S1 Table). To estimate $\lambda_{\mathrm{P},\epsilon}$, we fitted to following hierarchical model to our experimental data:

$$
\begin{aligned}
y_i &\sim \mathrm{Bernoulli}(\lambda) \quad , \\
\lambda &\sim \mathrm{Beta}(\mu\kappa, (1-\mu)\kappa) \quad , \\
\mu &\sim \mathrm{Beta}(1, 1) \quad , \\
\kappa &\sim \mathrm{HalfNormal}(10) \quad .
\end{aligned}
\tag{S1}
$$

This hierarchical model assumes that the number of predatory adults on each plate is drawn from a Bernoulli distribution with a rate that itself is drawn from a beta distribution.
