# Supplementary material for: Spatial and temporal heterogeneity alter the cost of plasticity in Pristionchus pacificus
Source: PLoS Comput Biol. 2024 Jan 30;20(1):e1011823. doi: 10.1371/journal.pcbi.1011823 (PMC10857712; doi:10.1371/journal.pcbi.1011823)
Supplement: S1 Table — (PDF) [file pcbi.1011823.s002.pdf]

S1 Table. Laboratory data on the mouth-form plasticity of *P. pacificus* RSC017 across two bacterial conditions.

Replicate	Strain	Diet	Predatory	Total
1	RSC017	*E. coli* OP50	1	50
2	RSC017	*E. coli* OP50	5	50
3	RSC017	*E. coli* OP50	1	50
1	RSC017	*Novosphingobium* sp. L76	44	50
2	RSC017	*Novosphingobium* sp. L76	47	50
3	RSC017	*Novosphingobium* sp. L76	42	50

Method: Each row indicates a plate with 50 adult worms of the plastic strain (RSC017), grown on one of the two alternative diets. A total of 300 worms were assayed.

Reference: M. Dardiry, V. Piskobulu, A. Kalirad, and R. J. Sommer. Experimental and theoretical support for costs of plasticity and phenotype in a nematode cannibalistic trait. *Evolution Letters*, 7(1):48–57, 01 2023.
